# Enriched Rehabilitation Improves Gait Disorder and Cognitive Function in Parkinson’s Disease: A Randomized Clinical Trial

**DOI:** 10.3389/fnins.2021.733311

**Published:** 2021-12-02

**Authors:** Xin Wang, LanLan Chen, Hongyu Zhou, Yao Xu, Hongying Zhang, Wenrui Yang, XiaoJia Tang, Junya Wang, Yichen Lv, Ping Yan, Yuan Peng

**Affiliations:** ^1^Department of Rehabilitation Medicine, Clinical Medical College, Yangzhou University, Yangzhou, China; ^2^Department of Neurology, Clinical Medical College, Yangzhou University, Yangzhou, China; ^3^Department of Medical Imaging, Clinical Medical College, Yangzhou University, Yangzhou, China; ^4^Graduate School, Dalian Medical University, Dalian, China; ^5^Medical College, Yangzhou University, Yangzhou, China; ^6^School of Rehabilitation Medicine, Binzhou Medical University, Yantai, China; ^7^School of Nursing, Yangzhou University, Yangzhou, China; ^8^Department of Rehabilitation Medicine, Guangzhou First People’s Hospital, Guangzhou, China

**Keywords:** enriched rehabilitation, cognitive function, gait disorder, Parkinson’s disease, left dorsolateral prefrontal cortex

## Abstract

**Background:** Studies on non-pharmacological strategies for improving gait performance and cognition in Parkinson’s disease (PD) are of great significance. We aimed to investigate the effect of and mechanism underlying enriched rehabilitation as a potentially effective strategy for improving gait performance and cognition in early-stage PD.

**Methods:** Forty participants with early-stage PD were randomly assigned to receive 12 weeks (2 h/day, 6 days/week) of enriched rehabilitation (ER; *n* = 20; mean age, 66.14 ± 4.15 years; 45% men) or conventional rehabilitation (CR; *n* = 20; mean age 65.32 ± 4.23 years; 50% men). In addition, 20 age-matched healthy volunteers were enrolled as a control (HC) group. We assessed the general motor function using the Unified PD Rating Scale—Part III (UPDRS-III) and gait performance during single-task (ST) and dual-task (DT) conditions pre- and post-intervention. Cognitive function assessments included the Montreal Cognitive Assessment (MoCA), the Symbol Digit Modalities Test (SDMT), and the Trail Making Test (TMT), which were conducted pre- and post-intervention. We also investigated alteration in positive resting-state functional connectivity (RSFC) of the left dorsolateral prefrontal cortex (DLPFC) in participants with PD, mediated by ER, using functional magnetic resonance imaging (fMRI).

**Results:** Compared with the HC group, PD participants in both ER and CR groups performed consistently poorer on cognitive and motor assessments. Significant improvements were observed in general motor function as assessed by the UPDRS-III in both ER and CR groups post-intervention. However, only the ER group showed improvements in gait parameters under ST and DT conditions post-intervention. Moreover, ER had a significant effect on cognition, which was reflected in increased MoCA, SDMT, and TMT scores post-intervention. MoCA, SDMT, and TMT scores were significantly different between ER and CR groups post-intervention. The RSFC analysis showed strengthened positive functional connectivity between the left DLPFC and other brain areas including the left insula and left inferior frontal gyrus (LIFG) post-ER.

**Conclusion:** Our findings indicated that ER could serve as a potentially effective therapy for early-stage PD for improving gait performance and cognitive function. The underlying mechanism based on fMRI involved strengthened RSFC between the left DLPFC and other brain areas (e.g., the left insula and LIFG).

## Introduction

Parkinson’s disease (PD) is the second most common neurodegenerative disorder in older adults, which is typically characterized by motor and non-motor impairments that lead to increasingly serious physical disability (Homayoun, 2018; Armstrong and Okun, 2020). Gait impairments remain the most common motor symptoms and are characterized by abnormal spatiotemporal variables, such as gait speed, stride length, and cadence (Creaby and Cole, 2018; Islam et al., 2020). Furthermore, cognitive deficits are prominently exhibited non-motor symptoms as the disease progresses (Goldman et al., 2018). However, the early onset of cognitive deficits can exacerbate gait disorder because cognition is vital for controlling both bilateral coordination and dynamic posture during walking (Lord et al., 2014; Morris et al., 2019). Clinical evidence has shown that gait deficits in PD patients are more obvious during dual-task (DT) conditions, which are more cognitively demanding owing to the simultaneous performance of cognitive and motor tasks, than during single-task (ST) conditions (Salazar et al., 2017). Despite emerging clinical targets for enhancing motor and cognitive functions simultaneously, pharmacological strategies, such as dopamine replacement therapy, usually only resolve motor symptoms while barely improving cognitive function (Armstrong and Okun, 2020). Therefore, research on non-pharmacological strategies for improving motor and cognitive outcomes in PD is highly warranted (Petzinger et al., 2013; Dagan et al., 2018; Ni et al., 2018).

Environmental enrichment is a viable non-pharmacological strategy in which participants are permitted to explore and interact with each other in a multistimuli environment to participate in highly social, physical, and cognitive activities simultaneously. Clinical and animal studies have shown that environmental enrichment has the potential to improve functional outcomes by triggering neuroplasticity in participants with neurological diseases, such as stroke and traumatic brain injury (Bondi et al., 2014; Wang et al., 2016, 2020). Although cognitive deficits have been shown to attenuate, motor impairments do not always show improvement by simple immersion in an enriched environment (McDonald et al., 2018). Therefore, conducting comprehensive training batteries is vital to synergistically improve behavioral outcomes in patients with neurological diseases (Schuch et al., 2016). A more effective paradigm that could dramatically promote overall function is enriched rehabilitation (ER), which is an integrated strategy that combines environmental enrichment with task-oriented training (Schuch et al., 2016; Vive et al., 2020). Evidence from animal studies has demonstrated that ER restores limb function better than does environment enrichment alone in patients with stroke (Schuch et al., 2016). Moreover, a recent clinical study reported a marked improvement in motor and cognitive function mediated by ER in chronic stroke patients (Vive et al., 2020). However, few studies have explored the effect of ER on PD gait disorders and cognitive function (Shah et al., 2016; Fujiwara et al., 2019). Furthermore, the specific mechanism by which ER enhances cognitive function remains unclear.

Resting-state functional magnetic resonance imaging (rs-fMRI) is an extensively used method for exploring neuro-rehabilitation mechanisms by detecting changes in blood oxygenation level-dependent intensity (Lv et al., 2018). To date, resting-state functional connectivity (RSFC) assessed by rs-fMRI has enabled the identification of the functional interaction between brain regions to understand the pathophysiological mechanisms underlying PD (Chen et al., 2020). Rs-fMRI studies have demonstrated that the left dorsolateral prefrontal cortex (DLPFC), one of the vital regions in the central executive network (CEN) that mediates cognitive function, has decreased functional connectivity with other brain areas in PD compared with healthy controls (Prodoehl et al., 2014; Caspers et al., 2017). Furthermore, studies on global connectivity have shown that improved cognition is related to stronger functional connectivity of the DLPFC with specific brain areas, such as the frontal, parietal, occipital, and limbic regions (Trujillo et al., 2015). Thus, we hypothesized that ER would improve gait disorder and cognitive function in early-stage PD patients by inducing neuroplasticity in the form of brain network changes. Therefore, the current study explored the effect of and mechanisms underlying ER in early-stage PD patients with gait disorder to assess its viability as a non-pharmacological strategy for PD rehabilitation.

## Materials and Methods

### Study Population

**FIGURE 1:**
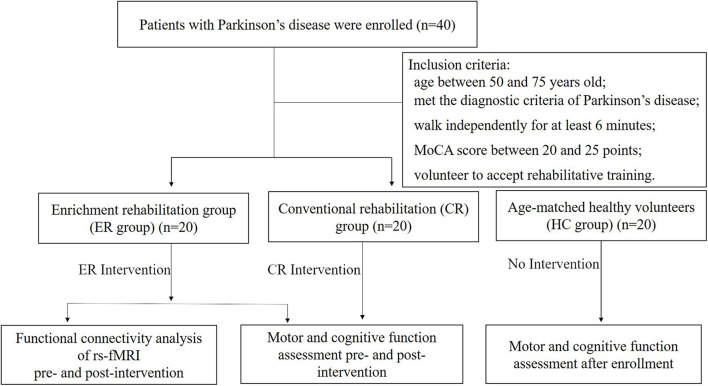
Flowchart illustrating the process for the study. MoCA, montreal cognitive assessment.

All participants were enrolled from the outpatient center at the Clinical College of Yangzhou University between September 2019 and October 2020. Participants with PD were recruited from the Department of Neurology and Rehabilitation. Inclusion criteria were as follows: (1) aged 50–75 years; (2) met diagnostic criteria for PD based on the United Kingdom Parkinson’s Disease Society Brain Bank, with confirmation made by two neurologists; (3) able to walk independently for at least 6 min (with rest intervals) without an assistive device; (4) an educational level higher than junior high school and a Montreal Cognitive Assessment (MoCA) score between 20 and 25 points; (5) severity of PD between grade 1 and 2 as assessed by the revised Hoehn–Yahr scale; (6) on a stable drug regimen for more than 2 weeks; and (7) volunteered to accept rehabilitative training. Exclusion criteria were as follows: (1) secondary or acquired parkinsonism; (2) other neurological disorders, such as stroke or previous traumatic brain injury; (3) severe cognitive impairment based on a MoCA score of < 20 points; (4) alcohol or drug abuse; (5) poor compliance with prescribed treatment; and (6) medical complications, such as severe lung and heart disease based on clinical symptoms and medical history. Based on these inclusion and exclusion criteria, 40 participants with PD were enrolled and randomly assigned into the enrichment rehabilitation (ER; *n* = 20) or conventional rehabilitation (CR; *n* = 20) group. In addition, 20 age-matched healthy volunteers were recruited from the Department of Physical Examination Center as the healthy control (HC) group. This study was approved by the Ethical Review Board of the Clinical Medical College in Yangzhou University, China (No. 2019070). All participants provided informed consent preintervention. Figure 1 describes the process of the study.

### Intervention

Participants in the ER and CR groups (i.e., PD groups) received ER and CR for 2 h/day, 6 days/week, for 12 consecutive weeks. Rehabilitative activities in the ER and CR groups were implemented by the same physical therapy team without knowing the aim of the study. A total of 65 therapists participated in the study. All rehabilitative activities were in mild to moderate intensity monitored by a wristwatch (Apple Watch Series 5) during the rehabilitation sessions, which induced a target heart rate below 65–70% of the maximum heart rate (Garber et al., 2011; Marusiak et al., 2019). Participants in the HC group received no intervention. We stopped other additional rehabilitation to avoid interferences with the treatment effect from the interventions used in this study.

Enriched rehabilitation was conceived and developed by combining environmental enrichment with repetitive and meaningful individual functional training during everyday tasks (Bondi et al., 2014; Vive et al., 2020). Participants in the ER group were unaware that the ER was not CR. The ER group was exposed to an enriched environment with easy access to both communal and individual equipment and activities for individual intensive rehabilitation (Vive et al., 2020). The rehabilitation training contents focused on three key areas, which are summarized below:

1. Enriched sensorimotor environmental stimulation paired with different types of sensory and motor exercises: to simulate the effect of an enriched environment, ER was carried out in a space with periodically changing light, sound, aroma, and lawn using multimedia equipment (Supplementary Figure 1). In addition, varied tactile textures and diverse thermal, visual, and auditory stimulation were provided as previous studies reported (Janssen et al., 2014). Integrated sensory and balance training was provided through playing games in virtual reality scenarios during 10-min exercise sessions to improve balance and coordination. Dynamic cycling training under different intensities and rhythms of music was also provided to improve motor function. Detailed information is listed in Supplementary Table 1.
2. Cognition-related activities integrated with specific tasks: participants were able to access interesting web pages by using an internet-connected computer and summarize the content in the ER area. Participants were also encouraged to read and recite their favorite books or articles in the electronic library. Simple board games and recreational activities (i.e., bingo) were made available throughout the intervention period, which need only single-player experiences on the tabletop to improve cognition. We provided individual enrichment by including music, audiobooks, and number puzzles.
3. Social interactions and therapeutic rehabilitation support: participants were encouraged to communicate with their family members about a particular topic with the guidance therapists. Participants in the ER group also interacted socially by participating in different activities together, such as playing board games (i.e., mahjong) and table tennis, which were cooperative and competitive activities to improve cognition and social skills in the meanwhile.

Participants in the CR group participated in standard physical therapy, which included balance and gait training (Ni et al., 2018). Balance training referred to usual static and dynamic balance activities, such as standing with one leg on stable or unstable surfaces and tilting the body in different directions to maximum angles in a seated or standing position. Gait training included bearing weight on the affected leg, walking on a treadmill, stepping onto a step, and walking on various types of surfaces. Detailed information on CR is provided in Supplementary Table 2.

### Outcomes and Measurement Procedures

Detailed demographic and medical characteristics, including age, sex, length of lower limbs, Hoehn–Yahr stage, symptom-dominant side, daily levodopa dosage, and disease duration, were recorded. For the PD groups, general motor function, gait performance, and cognition were assessed at two time points: (1) 72 h before the first rehabilitation training (pre-intervention) and (2) 24 h after the 12-week rehabilitation process (post-intervention). General motor function of participants with PD was assessed using the Unified PD Rating Scale—Part III (UPDRS-III; no disability 0, severe disability 108) and Hoehn–Yahr stage (minimal functional disability, confinement to wheelchair/bed) (Opara et al., 2017). To evaluate cognitive function, we administered the validated Chinese versions of the following scales: (1) the MoCA, which is used to assess general cognitive function, (2) the Symbol Digit Modalities Test (SDMT) to evaluate processing speed and attention, and (3) the Trail Making Test (TMT), which assesses the executive function (Federico et al., 2015). Motor and cognitive functions of the HC group were assessed within 24 h of enrollment. All assessments were carried out by a trained neuropsychologist who was blinded to patient group assignment.

Gait analysis of all participants was examined under ST and DT conditions. For both ST and DT, participants walked the length of a 20-m straight corridor that was free of obstacles at their preferred speed. In the DT condition, participants continuously performed a serial seven-subtraction task (i.e., 100−7 = 93, 93−7 = 86) while walking at their usual pace. Spatiotemporal gait parameters were acquired using wearable sensors of inertial measurement units (GYENNO Science, Shenzhen, China) (Brognara et al., 2019). Each participant was equipped with 10 sensors on the lower back, chest, bilateral feet, ankles, thighs, and wrists using elastic belts, which recorded the overall gait posture during walking (Cao et al., 2020). Each sensor collected real-time spatial and temporal gait information of the participants while walking, and the information was transmitted to a host computer *via* a Bluetooth link for further processing and storage. Gait parameters, including speed and stride length, were recorded. For each walking condition, repetitions were performed to ensure that three complete 10-s walking processes were recorded; the average values of the gait parameters were used for subsequent analyses. The first and last meters of the walking process were not included in the analysis of gait parameters to eliminate the effects of the acceleration and deceleration phases (Stuck et al., 2020).

### Magnetic Resonance Imaging Data Acquisition

To compare changes in functional connectivity mediated by ER, each patient in the ER group underwent brain magnetic resonance imaging (MRI), which included T1-weighted and rs-fMRI acquisitions, within 24 h pre- and post-intervention. T1-weighted images were used for reconstructing individual structural brain anatomy and were acquired using the following parameters: pulse repetition time/echo time = 1,900 ms/3.39 ms, field of view (FOV) = 240 × 176 mm, matrix = 256 × 176, slice thickness = 0.9375 mm, flip angle = 7°, reverse time = 1,100 ms, scan time = 4 min, and number of layers = 32. T2^∗^-weighted rs-fMRI volumes were used for functional connectivity analyses and were acquired using the following parameters: echo-planar imaging sequence = 31 slices, pulse repetition time/echo time = 2,000/30 ms, slice thickness = 4 mm, matrix = 64 × 64 mm, FOV = 240 × 240 mm, flip angle = 90°, number of layers = 31, and scanning time = 8 min. Each patient was asked to keep their eyes closed and stay as still as possible during the scan.

### Functional Connectivity Analysis

To qualitatively and quantitatively compare left DLPFC functional connectivity between post- and pre-ER, we preprocessed data using the Statistical Parametric Mapping 8 (SPM8) software (Wellcome Centre for Human Neuroimaging, London, United Kingdom) in MATLAB to prepare the rs-fMRI data. To exclude motion-related signals from the data, images with a maximum displacement of 3.0 mm in the x-, y-, or z-direction or 3° in any angle direction were discarded. All images were time shifted so that the slices were temporally aligned before slices were segmented into gray and white matter by co-registering with anatomical images. The Montreal Neurological Institute (MNI) template was applied to normalize the anatomical images, and the normalized parameters were used for the functional images. The linear trend was removed, and a temporal band-pass filter (0.01–0.08 Hz) was applied for regional homogeneity.

For the RSFC analysis, the Resting-state fMRI Data Analysis Toolkit was used for processing. Functional connectivity was measured using the seed-based correlation method using the CONN-fMRI functional connectivity toolbox. We measured the connectivity between the left DLPFC (MNI coordinates: *x* = −42, *y* = 33, *z* = 21) and each voxel of the brain. Correlations maps were calculated for each subject by extracting the mean signal time course from the seed and computing Pearson’s correlations coefficients with the time courses of all other voxels of the brain. These correlation coefficients were converted into normally distributed z-scores using the Fisher transformation to allow for linear model analyses.

### Statistical Analysis

Statistical analysis was performed using SPSS 22.0 (IBM Corp., Armonk, NY, United States). Data were described as means ± standard deviations (SDs). As coefficients of variation (CV) was considered a better indicator of gait performance, we calculated CV of gait performance for analysis as follows: CV = (standard deviation/mean) × 100%. A chi-square test was used to analyze the between-group differences for categorical variables, such as gender and symptom-dominant side. A one-way repeated-measures analysis of variance was performed to detect between-group differences (ER, CR, and HC groups) for continuous variables, which included motor and cognitive performance, and a least significant difference test was performed for multiple comparison correction. Independent t-test was conducted to compare the between-group differences post-intervention (ER and CR group) in continuous variables including motor and cognitive performance. Paired t-tests were used to detect within-group differences (pre- vs. post-intervention in the PD groups) for motor and cognitive performance. A statistical significance was set at *p* < 0.05.

Group-level statistical analyses of the fMRI data were performed using a random-effects model in SPM8. A two-sample t-test between groups (pre- vs. post-ER) was applied to the individual z-maps of the two groups, using small volume correction for the one-sample results masks. Multiple comparison correction was implemented using Monte Carlo simulation^1^. Significant between-group differences met the criteria of an uncorrected *p* < 0.01 at the voxel level and a cluster size of > 17 voxels, which corresponded to a corrected *p* < 0.05. To examine differences in alterations in functional connectivity, one-sample t-tests were used to compare individual within-group peak voxel z-maps, using a significance criterion of *p* < 0.05 in the SPSS 22.0 software package. Dissociable anomalies in functional connectivity patterns between groups (pre- vs. post-ER) were explored in the whole brain using a criterion of corrected *p* < 0.05 at the voxel level and a cluster size of > 228 voxels. The alpha for all significant results was two-tailed, except where noted.

## Results

### Demographics

There were no significant differences in baseline characteristics between the HC group and the PD groups, including age, sex, and length of lower limbs (Table 1, *p* > 0.05). Demographic characteristics, including age, gender, length of lower limbs, Hoehn–Yahr stage, symptom-dominant side, daily levodopa dosage, and disease duration were comparable between the ER and CR groups (Table 1, *p* > 0.05).

**TABLE 1:** Characteristics of the patients in the study groups.

**Characteristics**	**ER group (*n* = 20)**	**CR group (*n* = 20)**	**HC group (*n* = 20)**	***p***
Age (year)	65.25 ± 4.15	64.95 ± 3.99	66.15 ± 3.58	0.52
Sex, male/female	9/11	10/10	11/9	0.819
Hoehn and Yahr	1.37 ± 0.41	1.32 ± 0.36	–	0.395
Disease duration (year)	2.23 ± 1.69	2.28 ± 1.53	–	0.095
Symptom-dominant side (R/L)	12/8	12/8	–	1
Daily levodopa dosage (mg/day)	227 ± 116	230 ± 113	–	0.102
Length of lower limbs (cm)	83.76 ± 4.45	84.75 ± 4.82	83.99 ± 4.75	0.232

*ER, enriched rehabilitation; CR, conventional rehabilitation; HC, healthy control.*

### Behavioral Performance

#### Gait Parameters in the Single-Task and Dual-Task Conditions

As shown in Table 2, there were no significant differences in gait speed or stride length between the PD groups and the HC group pre-intervention under the ST condition (*p* > 0.05), whereas CVs of gait speed and stride length were significantly different (*p* < 0.05). Before the intervention, there were no significant differences in gait parameters between the PD groups under the ST condition (*p* > 0.05). The CVs of gait speed and stride length significantly improved in the ER group post-intervention (*p* < 0.05). In addition, significant differences were observed in the CVs of gait speed and stride length between the ER and CR groups in the ST condition post-intervention (*p* < 0.05).

**TABLE 2:** Gait characteristics for all participants pre- and post-intervention.

	**ST**					**DT**				
	**ER group (*n* = 20)**	**CR group (*n* = 20)**	**HC group (*n* = 20)**	**F^**	***p^***	**ER group (*n* = 20)**	**CR group (*n* = 20)**	**HC group (*n* = 20)**	**F^**	***p^***
**Gait speed (m/s)**										
Pre-intervention	1.17 ± 0.08	1.16 ± 0.10	1.19 ± 0.05	0.56	0.53	0.79 ± 0.11*	0.76 ± 0.16*	1.09 ± 0.08^&^	46.67	0.40
Post-intervention	1.18 ± 0.07	1.17 ± 0.08		0.91	0.37	0.91 ± 0.15^*#$^	0.78 ± 0.11*		3.17	0.00
**CV of gait speed (%)**										
Pre-intervention	6.67 ± 1.58*	6.65 ± 1.23*	4.42 ± 1.19^&^	17.66	0.96	15.46 ± 3.33*	15.62 ± 3.71*	8.24 ± 1.01^&^	39.11	0.87
Post-intervention	5.13 ± 0.92^*#$^	6.76 ± 1.13*		−4.87	0.00	12.81 ± 3.14^*#$^	15.48 ± 3.69*		−2.41	0.00
**Stride length (m)**										
Pre-intervention	1.13 ± 0.13	1.11 ± 0.13	1.11 ± 0.07	0.15	0.72	0.82 ± 0.11*	0.83 ± 0.18*	1.03 ± 0.09^&^	14.24	0.87
Post-intervention	1.14 ± 0.09	1.09 ± 0.13		1.46	0.15	0.92 ± 0.08^*#$^	0.84 ± 0.14*		2.46	0.01
**CV of stride length (%)**										
Pre-intervention	9.74 ± 2.14*	9.94 ± 2.53*	6.12 ± 1.93^&^	17.99	0.77	15.06 ± 4.78*	15.29 ± 4.16*	7.79 ± 1.05^&^	25.15	0.85
Post-intervention	7.11 ± 1.99^*#$^	9.92 ± 2.31*		−4.03	0.00	11.33 ± 3.58^*#$^	15.32 ± 4.20*		−3.15	0.00

*ST, single-task; DT, dual-task; ER, enriched rehabilitation; CR, conventional rehabilitation; HC, healthy control.*

*Compared with HC group pre-intervention, *p* < 0.05.

*^&^Compared with CR group pre-intervention, *p* < 0.05.*

*^#^Compared with CR group post-intervention, *p* < 0.05.*

*^$^Compared with pre-intervention, *p* < 0.05.*

*F^, the *F*-value of ER group compared with CR group; *p*^, the *p*-value of ER group compared with CR group.*

As indicated in Table 2, under the DT condition, gait parameters of the ER and CR groups were significantly different from those of the HC group pre-intervention (*p* < 0.05). There were no significant differences in gait parameters between the PD groups pre-intervention under the DT condition (*p* > 0.05). No significant changes in gait parameters were observed in the CR group post-intervention (*p* > 0.05). However, the gait parameters of gait speed, stride length, CV of gait speed, and CV of stride length under the DT condition of the ER group improved post-intervention (*p* < 0.05). Moreover, significant differences were observed in gait parameters under the DT condition between the ER and CR groups post-intervention (*p* < 0.05).

#### Motor and Cognitive Outcomes

There was a significant change from pre- to post-intervention in both PD groups for UPDRS-III scores (Table 3; *p* < 0.05), which indicated that the PD groups performed consistently better overall during motor assessments following the interventions. There was no significant difference in UPDRS-III score between the PD groups post-intervention (*p* > 0.05), which indicated that there were no differential effects of the interventions on general motor function.

**TABLE 3:** Motor and cognitive assessments for all participants pre- and post-intervention.

	**ER group (*n* = 20)**	**CR group (*n* = 20)**	**HC group (*n* = 20)**	**F^**	***p*^**
**UPDRS-III**					
Pre-intervention	15.35 ± 2.83	15.25 ± 2.12		0.12	0.90
Post-intervention	10.95 ± 2.56^$^	11.15 ± 1.65^$^		−0.29	0.78
**MoCA**					
Pre-intervention	23.05 ± 1.28*	23.15 ± 1.24*	29.15 ± 0.48^&^	204.44	0.77
Post-intervention	26.15 ± 1.93^*#$^	23.55 ± 1.56*		4.56	0.00
**SDMT**					
Pre-intervention	48.85 ± 10.25*	48.15 ± 10.29*	73.25 ± 7.48^&^	63.64	0.82
Post-intervention	64.15 ± 10.03^*#$^	47.55 ± 11.86*		4.66	0.00
**TMT-A (s)**					
Pre-intervention	73.68 ± 18.75*	72.54 ± 18.22*	48.12 ± 12.09^&^	14.33	0.83
Post-intervention	72.72 ± 18.36*	72.85 ± 12.78*		−0.02	0.98
**TMT-B (s)**					
Pre-intervention	148.60 ± 34.44*	148.67 ± 33.57*	99.17 ± 19.95^&^	17.15	0.99
Post-intervention	125.80 ± 29.65^*#$^	150.62 ± 37.14*		−2.28	0.03

ER, enriched rehabilitation; CR, conventional rehabilitation; HC, healthy control. *Compared with HC group pre-intervention, *p* < 0.05.

*^&^Compared with CR group pre-intervention, *p* < 0.05.*

*^#^Compared with CR group post-intervention, *p* < 0.05.*

*^$^Compared with pre-intervention, *p* < 0.05.*

*F^, the *F*-value of ER group compared with CR group; *p*^, the *p*-value of ER group compared with CR group.*

The cognitive assessment results of each PD group pre- and post-intervention are summarized in Table 3. Cognitive function scores of the MoCA, SDMT, and TMT were significantly different between the PD groups and HC group pre-intervention (*p* < 0.05); however, they did not differ significantly between the PD groups (*p* > 0.05), which indicated that PD participants performed consistently poorer on the cognitive assessments than did the HC group at baseline. No changes were observed in cognitive scores from pre- to post-intervention in the CR group (*p* > 0.05), which indicated that CR had no effect on cognition. In contrast, there was a significant therapeutic effect of ER on cognition, which was shown by increased MoCA, SDMT, and TMT scores from pre- to post-intervention (*p* < 0.05). Moreover, these cognitive function scores were significantly different between the PD groups post-intervention (*p* < 0.05).

#### Functional Connectivity Analysis

**FIGURE 2:**
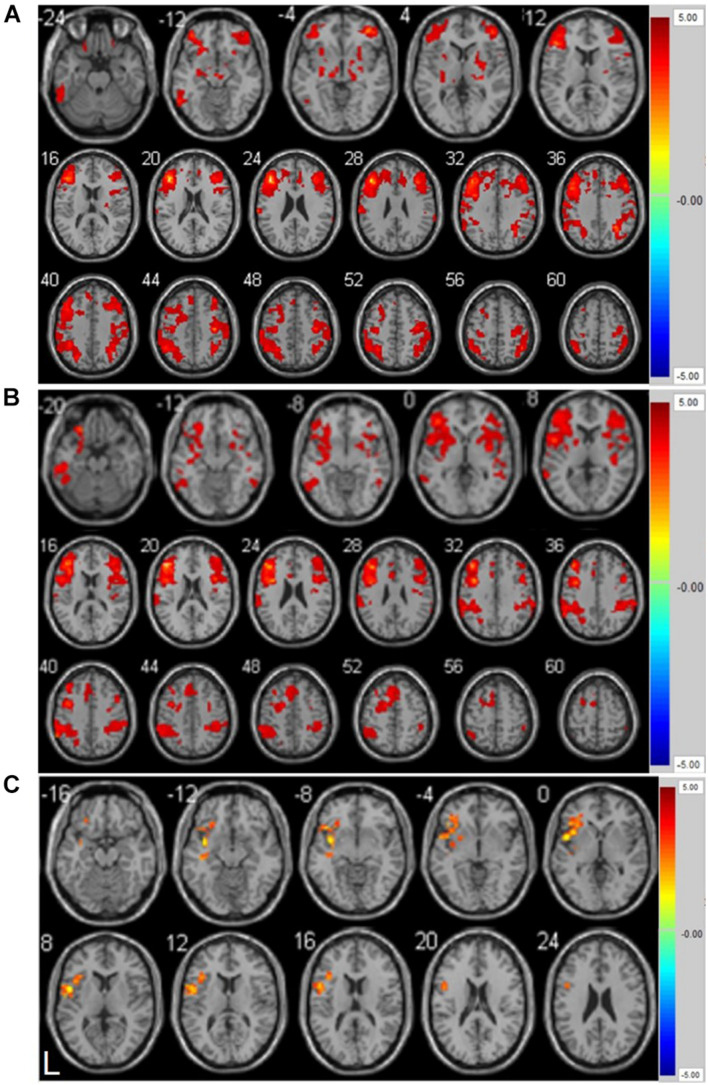
**(A)** Positive resting-state functional connectivity with left DLPFC pre-ER intervention in PD patients. **(B)** Positive resting-state functional connectivity with left DLPFC post-ER intervention in PD patients. **(C)** Differential positive resting-state functional connectivity with left DLPFC between pre- and post-ER intervention in PD patients (*p* < 0.05, Alphasim corrected, cluster size > 228 voxels). Abbreviation: ER, enriched rehabilitation.

As shown in Figure 2A and Supplementary Table 5, the left inferior temporal gyrus, bilateral middle frontal gyrus, right lenticular putamen, and right precentral gyrus showed positive functional connectivity with the left DLPFC pre-ER (*p* < 0.05, cluster size > 228 voxels). Supplementary Table 6 and Figure 2B show that the left inferior temporal gyrus, left middle frontal gyrus, right triangular inferior frontal gyrus (IFG), left parietal inferior marginal angular gyrus, and right superior marginal gyrus exhibited positive RSFC with the left DLPFC post-ER (*p* < 0.05, cluster size > 228 voxels). As shown in Figure 2C and Table 4, RSFC between the left DLPFC and deep brain regions, including the left insula and left IFG (LIFG), was stronger post-ER compared with pre-ER (*p* < 0.05, Alphasim corrected, cluster size > 228 voxels).

**TABLE 4:** Strengthened positive resting-state functional connectivity (RSFC) of left DLPFC post- compared with pre-ER intervention in PD patients.

**Region**	**BA**	**MNI coordinate (mm)**	**Peak *T* value**	**Cluster size**
		(X Y Z)		
Left insular cortex	13	−48 6 3	6.0982	216
Left inferior frontal gyrus	13/47	−32 20 6	8.0220	164

*BA, Brodman’s area; cluster size, the number of voxels; MNI, Montreal Neurological Institute.*

*p < 0.05, Alphasim corrected, cluster size > 228 voxels.*

## Discussion

In this study, we investigated the effect of ER on gait performance and cognitive function in patients with PD. We compared the differentially positive RSFC of the left DLPFC before and after ER. The study yielded three main results: first, ER induced significant improvements in motor and walking function, as indexed by gait parameters in the ST and DT conditions. Second, ER training led to an improvement in cognitive function, which was shown by increases in MoCA, SDMT, and TMT scores following the intervention. ER had obvious advantage to improve cognition compared with CR. Third, we found a strengthened positive RSFC between the left DLPFC and the left insula and LIFG after ER, which suggested that ER induced neuroplasticity to restore cognitive and walking function in early-stage PD.

Walking is a complex task, whereby gait performance relies on the interplay between motor control and cognition (Forte et al., 2019; Wollesen et al., 2019). However, both motor and cognitive function are impaired even in the early stages of PD due to dopaminergic neuronal loss in the basal ganglia (San Martín Valenzuela et al., 2020). Consistent with previous studies, the PD groups in our study performed consistently poorer on cognitive and motor assessments than did the HC group (Opara et al., 2017; Szeto et al., 2020). The early onset of cognitive deficits exacerbates gait impairments in PD because attention and executive function are indispensable for maintaining gait performance, which includes bilateral coordination and dynamic posture (Ehgoetz Martens et al., 2018). Deleterious changes in the gait parameters of speed, stride width, and gait variability have been reported in DT studies of PD (Penko et al., 2018). As expected, lower gait speed and stride length were observed in the PD groups than in the HC group during the DT condition. However, no significant differences were seen in gait speed and stride length between the PD groups and the HC group during the ST condition. Previous studies have shown that the increased CV of gait parameters was related with a decrease in postural control ability, which is a strong predictor of fall (Hausdorff, 2009). Notably, we observed greater gait variability, including CV of gait speed and stride length, in the PD groups compared with that of the HC during ST and DT conditions. CV is likely more sensitive than gait speed and stride length for detecting gait disorder in relatively early stages of PD (Noh et al., 2020).

Neurorehabilitation is a potent intervention for improving motor function in patients with PD (Ekker et al., 2016; Ferrazzoli et al., 2020). A few studies have reported the potential of CR for PD, which include exercise and physical therapy, to alleviate motor deficits in muscle strength, balance, and endurance by targeting neuroplasticity (Mak et al., 2017; van der Kolk et al., 2019). It is also encouraging that rehabilitation (both the ER and CR groups) showed consistent improvement in general motor function as assessed by the UPDRS-III. However, our gait analysis showed that CR did not improve walking function in early-stage PD, especially during the DT condition, which is likely due to its lack of impact on cognitive function and, in turn, the lack of interaction between gait and cognition. Few studies have focused on rehabilitation strategies to simultaneously improve cognitive and motor functions, which would offer a better treatment protocol for PD patients in early-stage PD.

Environmental rehabilitation is a novel strategy to simultaneously ameliorate motor and cognitive deficits by combining environmental enrichment with task-specific therapy (Vive et al., 2020). Studies have confirmed the effect of ER on the improvement of motor-cognitive function in patients with stroke and neonatal hypoxia–ischemia (Schuch et al., 2016; Vive et al., 2020). In the present study, significant improvement was observed in walking function as assessed by gait parameters, accompanied by enhanced cognition post-ER in early-stage PD patients. Moreover, we revealed that ER was favorable over CR for improving walking function, especially under the DT condition, which was primarily related to the significant effect of ER on cognition. This conclusion is consistent with previous evidence, which reported a possible mechanism whereby brain cognitive framework changes caused by ER mediates neuroplasticity. We speculated that task-oriented exercise training in ER requires the activation of motor control and the attention-executive network simultaneously, which could strengthen the integration of motor and cognitive resources in turn. Besides, attention-executive function of PD patients was also enhanced by activities in ER including multisensory and social interactive stimulations, such as supermarket shopping and chess. However, the specific mechanism by which ER enhances cognitive function remains to be elucidated.

Our rs-fMRI analysis provided a quantitative metric of RSFC, which reflects the neural activity and functional integrity of the brain frameworks related to cognition (Lv et al., 2018). The left DLPFC is one of the vital regions within the CEN that mediates cognitive function including attention, working memory, and decision-making (Dong et al., 2020). Neuroimaging studies in participants with early-stage PD have demonstrated that the left DLPFC has decreased connectivity with the left insula (de Bondt et al., 2016), which is a vital regulatory brain area required for performing cognitive tasks (Fu et al., 2021). The involvement of the insula in cognitive tasks is primarily reflected in the flexible switching of attention, the regulation of goal-directed behavior, and the inhibition of irrelevant stimuli (Varjačić et al., 2018). Our data confirmed a strengthened positive RSFC between the left DLPFC and left insula in participants with PD, which was brought about by an enriched sensorimotor environmental stimulation paired with different types of sensory and motor exercises, alongside cognition-related activities and social interaction. In addition, the left IFG is pivotal for successfully exercising inhibitory control over motor responses (Leite et al., 2018). Studies showed that DLPFC and IFG play a complementary and dissociable role in the settlement of decision conflict (Mitchell et al., 2009). Moreover, our FC analysis demonstrated a strengthened positive FC between the left DLPFC and IFG after ER. We inferred from these findings that ER induces neuroplasticity in participants with early-stage PD through strengthening the FC between the left DLPFC and deep brain regions, including the left insular cortex and IFG, which resulted in a positive executive functional network to improve cognitive and walking function. Our findings demonstrated that ER has potential in the improvement of motor function and cognition in early-stage PD patients.

This study has several strengths. We analyzed gait parameters in both the ST and DT walking conditions in early-stage PD patients. Additionally, we investigated RSFC between the left DLPFC and other brain areas pre- and post-ER. However, there are also several limitations. First, the sample size in our study was small, and participants were recruited from only one hospital, which may have introduced bias. In addition, we did not stratify participants based on different clinical classifications of PD, which would likely impact the effect of ER. Furthermore, we only observed the RSFC alteration of PD patients induced by ER; the differential functional connectivity induced by ER and CR would be explored in our future study.

## Conclusion

Our findings indicated that ER has potential as a potent therapy for participants with early-stage PD for improving cognitive function and gait disorder. The underlying mechanism deduced from rs-fMRI was related to the intervention-induced neuroplasticity whereby the functional connectivity between the left DLPFC and the left insula and IFG was strengthened.

## Data Availability Statement

The original contributions presented in the study are included in the article/Supplementary Material, further inquiries can be directed to the corresponding authors.

## Ethics Statement

The studies involving human participants were reviewed and approved by Ethical Review Board of Clinical Medical College of Yangzhou University, China (No. 2019070). All study subjects were recruited by the Clinical Medical College of Yangzhou University. The patients/participants provided their written informed consent to participate in this study.

## Author Contributions

XW, LC, and YP designed the study. XW, LC, HZo, YX, HZa, and WY performed the experiments. XW, XT, JW, YL, PY, and YP analyzed the data. XW, LC, and YP wrote the manuscript. All authors contributed to the article and approved the submitted version.

## Conflict of Interest

The authors declare that the research was conducted in the absence of any commercial or financial relationships that could be construed as a potential conflict of interest.

## Publisher’s Note

All claims expressed in this article are solely those of the authors and do not necessarily represent those of their affiliated organizations, or those of the publisher, the editors and the reviewers. Any product that may be evaluated in this article, or claim that may be made by its manufacturer, is not guaranteed or endorsed by the publisher.

## Footnotes

1
http://afni.nimh.nih.gov/pub/dist/doc/manual/AlphaSim.pdf
