# Supplementary figures and images for: Enriched Rehabilitation Improves Gait Disorder and Cognitive Function in Parkinson’s Disease: A Randomized Clinical Trial
Source: Front Neurosci. 2021 Dec 2;15:733311. doi: 10.3389/fnins.2021.733311 (PMC8674725; doi:10.3389/fnins.2021.733311)

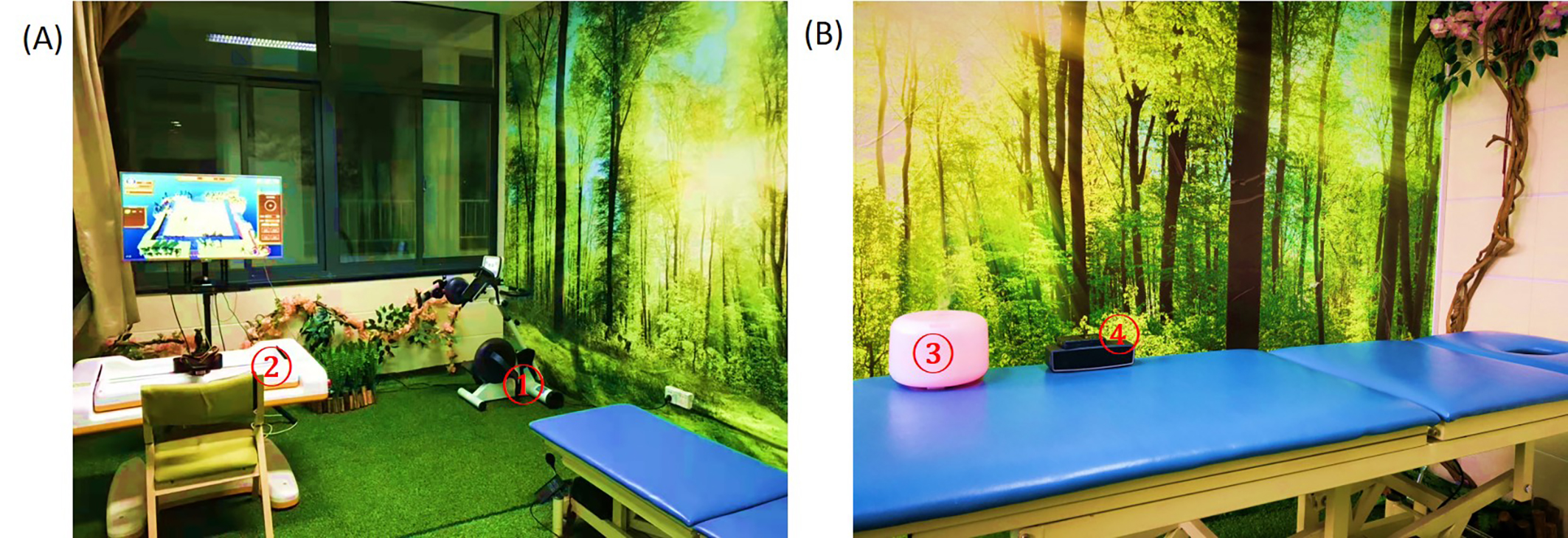

Supplement: Supplementary file 1 [file Image_1.TIF]
