# Supplementary material for: Enriched Rehabilitation Improves Gait Disorder and Cognitive Function in Parkinson’s Disease: A Randomized Clinical Trial
Source: Front Neurosci. 2021 Dec 2;15:733311. doi: 10.3389/fnins.2021.733311 (PMC8674725; doi:10.3389/fnins.2021.733311)
Supplement: Supplementary file 2 [file Table_1.doc]

Table S1 Intervention exercise for enriched rehabilitation

| Modality | Exercises | Details of exercises | Time (min) | Intensity |
| --- | --- | --- | --- | --- |
| Enriched environment with sensory and motor stimulation | Audio-visual olfactory stimulation | changed light, sound, aroma, and lawn using multimedia equipment | 15 | N/A |
|  | varied tactile textures, different thermal stimulation, various visual stimulation, and dives auditory stimulations | Touching objects of different textures while blindfolded, such as blankets, glass, etc  Touching a cup of water with hand at different temperatures  Watching TV or listening to music by PC |  | N/A |
|  | balance  training | Using VR games for balance training with weight shifts, such as picking mushroom，picking up different things on the floor, etc | 10 | 55~60%HRmax |
|  | motor stimulation | Dynamic cycling training with soothing and stirring music | 25 | 55~60%HRmaxfor 10 min  65~70%HRmax for 15 min |
| Cognitive stimulation | Cognition-related activities integrated with specific tasks | Browsing interesting web pages by using an internet-connected computer and then summarize the contents briefly;  Reading and reciting the favorite books or articles in the electronic library;  Recreational activities: Simple board games by PC, audiobooks, number puzzles,etc. | 30 | N/A |
| Social stimulation | Social interaction and therapeutic rehabilitation support | Communicating with their family members about a particular topic;  Interacted socially by joining different activities such as playing board games that is played by many people (majong), table tennis with each other, shopping at the supermarket with your family on a mission, etc | 40 | N/A |

Table S2 Intervention exercise for conventional rehabilitation

| Modality | Exercises | Time (min) | Intensity |
| --- | --- | --- | --- |
| Warm-up | Move the joints and stretch the musle of upper and lower limbs | 5 | 55~60%HRmax |
| Balance | Standing on one leg  Stable and unstable surfaces  Tilting the body in different directions at maximum angles in the seat and standing position | 25 | 55~60%HRmax |
| Gait training | relearn how to walk and improve walking patterns | 35 | 55~60%HRmax |
|  | walking on a treadmill | 15 | 65~70%HRmax |
|  | stepping onto a stair | 15 | 55~60%HRmax |
|  | walking over different types of surfaces | 15 | 55~60%HRmax |
|  | bearing weight on the affected leg | 10 | 55~60%HRmax |

Table S3 F value, freedom degrees and effect sizes of gait characteristics for all participants pre- and post-intervention

ER:enriched rehabilitation; CR:conventional rehabilitation; HC:healthy control; Pre-:pre-intervention; Post-: post-intervention; ST: single-task; DT:dual-task;

| gait speed in ST | | | | | | |
| --- | --- | --- | --- | --- | --- | --- |
|  | ER Pre- vs CR Pre- | ER Pre- vs HC | CR Pre- vs HC | ER Post- vs CR Post- | ER Post- vs ER Pre- | CR Post- vs CR Pre- |
| Freedom degree | (2, 57) | (2, 57) | (2, 57) | 38 | 19 | 19 |
| F/T value | 0.561 | 0.561 | 0.561 | 0.909 | 0.127 | 0.093 |
| effect size | 0.016 | 0.011 | 0.027 | 0.022 | 0.0025 | 0.003 |
| P | 0.53 | 0.675 | 0.297 | 0.369 | 0.136 | 0.927 |
| CV of gait speed in ST | | | | | | |
|  | ER Pre- vs CR Pre- | ER Pre- vs HC | CR Pre- vs HC | ER Post- vs CR Post- | ER Post- vs ER Pre- | CR Post- vs CR Pre- |
| Freedom degree | (2, 57) | (2, 57) | (2, 57) | 38 | 19 | 19 |
| F/T value | 17.663 | 17.663 | 17.663 | -4.874 | 3.723 | 0.293 |
| effect size | 0.02 | 2.255 | 2.234 | -1.622 | 1.5375 | 0.105 |
| P | 0.963 | 0 | 0 | 0 | 0.001 | 0.773 |
| stride length in ST | | | | | | |
|  | ER Pre- vs CR Pre- | ER Pre- vs HC | CR Pre- vs HC | ER Post- vs CR Post- | ER Post- vs ER Pre- | CR Post- vs CR Pre- |
| Freedom degree | (2, 57) | (2, 57) | (2, 57) | 32.872 | 19 | 19 |
| F/T value | 0.148 | 0.148 | 0.148 | 1.462 | -0.515 | 0.641 |
| effect size | 0.0125 | 0.018 | 0.006 | 0.052 | -0.0155 | 0.024 |
| P | 0.72 | 0.596 | 0.864 | 0.153 | 0.612 | 0.529 |
| CV of stride length in ST | | | | | | |
|  | ER Pre- vs CR Pre- | ER Pre- vs HC | CR Pre- vs HC | ER Post- vs CR Post- | ER Post- vs ER Pre- | CR Post- vs CR Pre- |
| Freedom degree | (2, 57) | (2, 57) | (2, 57) | 38 | 19 | 19 |
| F/T value | 17.992 | 17.992 | 17.992 | -4.025 | 3.267 | 0.029 |
| effect size | 0.209 | 3.621 | 3.829 | -2.812 | 2.628 | 0.0245 |
| P | 0.773 | 0 | 0 | 0.001 | 0.004 | 0.977 |
| gait speed in DT | | | | | | |
|  | ER Pre- vs CR Pre- | ER Pre- vs HC | CR Pre- vs HC | ER Post- vs CR Post- | ER Post- vs ER Pre- | CR Post- vs CR Pre- |
| Freedom degree | (2, 57) | (2, 57) | (2, 57) | 38 | 19 | 19 |
| F/T value | 46.665 | 46.665 | 46.665 | 3.173 | -3.149 | -0.292 |
| effect size | 0.033 | 0.301 | 0.334 | 0.1335 | -0.1165 | -0.0155 |
| P | 0.397 | 0 | 0 | 0.003 | 0.005 | 0.773 |
| CV of gait speed in DT | | | | | | |
|  | ER Pre- vs CR Pre- | ER Pre- vs HC | CR Pre- vs HC | ER Post- vs CR Post- | ER Post- vs ER Pre- | CR Post- vs CR Pre- |
| Freedom degree | (2, 57) | (2, 57) | (2, 57) | 38 | 19 | 19 |
| F/T value | 39.113 | 39.113 | 39.113 | -2.4 | 2.525 | 0.099 |
| effect size | 0.161 | 7.221 | 7.381 | -2.67 | 2.6465 | 0.137 |
| P | 0.867 | 0 | 0 | 0.021 | 0.021 | 0.922 |
| stride length in DT | | | | | | |
|  | ER Pre- vs CR Pre- | ER Pre- vs HC | CR Pre- vs HC | ER Post- vs CR Post- | ER Post- vs ER Pre- | CR Post- vs CR Pre- |
| Freedom degree | (2, 57) | (2, 57) | (2, 57) | 30.346 | 19 | 19 |
| F/T value | 14.238 | 14.238 | 14.238 | 2.458 | 2.699 | -0.115 |
| effect size | 0.007 | 0.205 | 0.197 | 0.0058 | 0.103 | -0.005 |
| P | 0.873 | 0 | 0 | 0.02 | 0.014 | 0.91 |
| CV of stride length in DT | | | | | | |
|  | ER Pre- vs CR Pre- | ER Pre- vs HC | CR Pre- vs HC | ER Post- vs CR Post- | ER Post- vs ER Pre- | CR Post- vs CR Pre- |
| Freedom degree | (2, 57) | (2, 57) | (2, 57) | 38 | 19 | 19 |
| F/T value | 25.147 | 25.147 | 25.147 | -3.152 | 3.073 | -0.023 |
| effect size | 0.234 | 7.268 | 7.501 | -3.988 | 3.724 | -0.03 |
| P | 0.847 | 0 | 0 | 0.003 | 0.006 | 0.982 |

Table S4 F value, freedom degrees and effect sizes of Motor and Cognitive Assessments for all participants pre- and post-intervention

ER:enriched rehabilitation; CR:conventional rehabilitation; HC:healthy control; Pre-:pre-intervention; Post-: post-intervention

| Assessment of UPRDS | | | | | | |
| --- | --- | --- | --- | --- | --- | --- |
|  | ER Pre- vs CR Pre- | ER Pre- vs HC | CR Pre- vs HC | ER Post- vs CR Post- | ER Post- vs ER Pre- | CR Post- vs CR Pre- |
| Freedom degree | 38 | / | / | 38 | 19 | 19 |
| F/T value | 0.123 | / | / | -0.286 | 6.985 | 7.216 |
| effect size | 0.1 |  |  | -0.2 | 4.4 | 4.1 |
| P | 0.903 |  |  | 0.776 | 0.001 | 0.001 |
| Assessment of MoCA | | | | | | |
|  | ER Pre- vs CR Pre- | ER Pre- vs HC | CR Pre- vs HC | ER Post- vs CR Post- | ER Post- vs ER Pre- | CR Post- vs CR Pre- |
| Freedom degree | (2, 57) | (2, 57) | (2, 57) | 38 | 19 | 19 |
| F/T value | 204.435 | 204.435 | 204.435 | 4.561 | -5.767 | -0.715 |
| effect size | 0.1 | 6.1 | 6 | 2.6 | -3.1 | -0.4 |
| P | 0.773 | 0 | 0 | 0.001 | 0.001 | 0.483 |
| Assessment of SDMT | | | | | | |
|  | ER Pre- vs CR Pre- | ER Pre- vs HC | CR Pre- vs HC | ER Post- vs CR Post- | ER Post- vs ER Pre- | CR Post- vs CR Pre- |
| Freedom degree | (2, 57) | (2, 57) | (2, 57) | 38 | 19 | 19 |
| F/T value | 43.642 | 43.642 | 43.642 | 4.66 | -4.732 | 0.186 |
| effect size | 0.7 | 24.4 | 25.1 | 16.6 | -15.3 | 0.6 |
| P | 0.82 | 0 | 0 | 0.001 | 0.001 | 0.854 |
| Assessment of TMT-A | | | | | | |
|  | ER Pre- vs CR Pre- | ER Pre- vs HC | CR Pre- vs HC | ER Post- vs CR Post- | ER Post- vs ER Pre- | CR Post- vs CR Pre- |
| Freedom degree | (2, 57) | (2, 57) | (2, 57) | 38 | 19 | 19 |
| F/T value | 14.327 | 14.327 | 14.327 | -0.021 | 0.148 | -0.05 |
| effect size | 1.139 | 25.562 | 24.423 | -0.125 | 0.9565 | -0.3075 |
| P | 0.834 | 0 | 0 | 0.984 | 0.884 | 0.96 |
| Assessment of TMT-B | | | | | | |
|  | ER Pre- vs CR Pre- | ER Pre- vs HC | CR Pre- vs HC | ER Post- vs CR Post- | ER Post- vs ER Pre- | CR Post- vs CR Pre- |
| Freedom degree | (2, 57) | (2, 57) | (2, 57) | 38 | 19 | 19 |
| F/T value | 17.15 | 17.15 | 17.15 | -2.277 | 2.088 | -0.174 |
| effect size | 0.072 | 49.435 | 49.506 | -24.8225 | 22.7985 | -1.9525 |
| P | 0.994 | 0 | 0 | 0.029 | 0.048 | 0.864 |

**Table S5** Positive Resting-State Functional Connectivity with left DLPFC pre-ER intervention in PD

| Brain Region | BA | MNI coordinate (mm) | Peak T value | Cluster size |
| --- | --- | --- | --- | --- |
|  |  | (X Y Z) |  |  |
| Left inferior temporal gyrus | 20/37 | -57 -54 -15 | 9.1005 | 248 |
| Left middle frontal gyrus | 40 | -42 33 24 | 27.4168 | 3312 |
| Right middle frontal gyrus | 9 | 42 45 -6 | 22.8629 | 1342 |
| Right lenticular putamen | 47 | -27 -15 -15 | 8.3363 | 371 |
| Right anterior central gyrus | 40 | 39 -18 45 | 14.5609 | 1147 |

BA, Brodman’s area; Cluster size, the number of voxels; MNI, Montreal Neurological Institute

P < 0.05, cluster size > 228 voxels.

**Table S6** Positive Resting-State Functional Connectivity with left DLPFC post-ER intervention in PD

| Brain Region | BA | MNI coordinate (mm) | Peak T value | Cluster size |
| --- | --- | --- | --- | --- |
|  |  | (X Y Z) |  |  |
| Left inferior temporal gyrus | 20 | -60 -27 -24 | 13.9607 | 504 |
| Left frontal lobe | 6 | -45 33 21 | 27.7504 | 572 |
| Left insula | 13 | -45 25 8 | 17.3987 | 250 |
| Right inferior frontal gyrus | 46 | 42 9 21 | 11.5647 | 330 |
| Left angular gyrus | 40 | -60 -54 39 | 13.3393 | 779 |

BA, Brodman’s area; Cluster size, the number of voxels; MNI, Montreal Neurological Institute

P < 0.05, cluster size > 228 voxels.

supplementary figure legend

Photos for Enriched rehabilitation training room

A: ①dynamic cycling; ② virtual reality scenarios;

B: ③ odor eliminators; ④music player
